# Supplementary material for: Neutral and Selective Processes Shape MHC Diversity in Roe Deer in Slovenia
Source: Animals (Basel). 2022 Mar 13;12(6):723. doi: 10.3390/ani12060723 (PMC8944837; doi:10.3390/ani12060723)
Supplement: Supplementary file 1 [file animals-12-00723-s001.zip › animals-1475938_Supplementary material_Figures.pdf]

Supplementary material. Figures

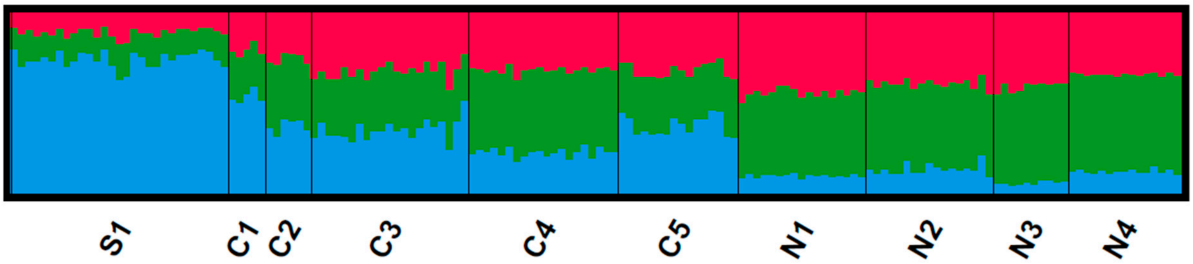

Figure S1. Genetic structure (from the STRUCTURE) of Slovene roe deer from 10 sampling areas/populations, based on microsatellites (for details on each individual, see Table S1). Each individual is represented by a line proportionally partitioned into colour segments corresponding to its membership in particular clusters (K = 3). Black lines separate individuals from different areas.

a)

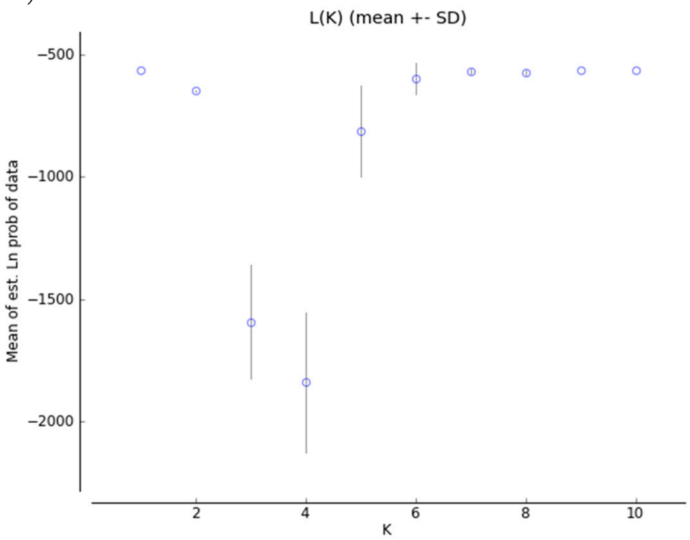

b)

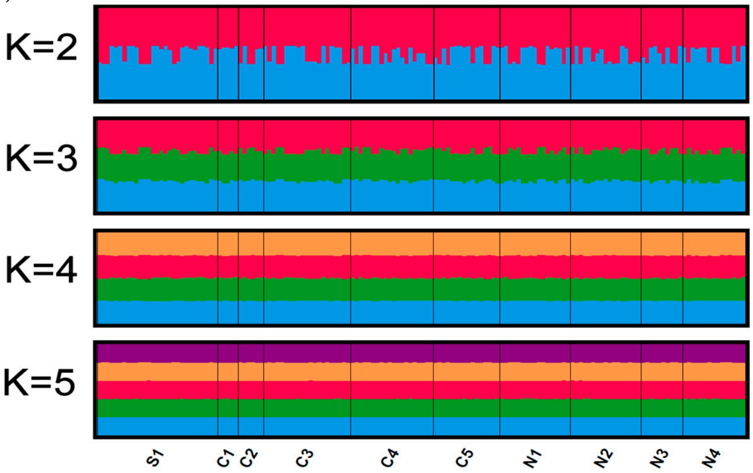

Figure S2. Results from the STRUCTURE analysis: (**a**) The highest natural log likelihood from STRUCTURE HARVESTER [103] suggests that the number of genetic groups (K) is most likely 1 when considering MHC DRB exon 2 alleles in roe deer female yearlings across Slovenia. (**b**) Analysis for different number of clusters (K = 2–5) of Slovene roe deer, based on MHC (for details on each individual, see Table S1). Each individual is represented by a line proportionally partitioned into colour segments corresponding to its membership in particular clusters. Black lines separate individuals from different areas.

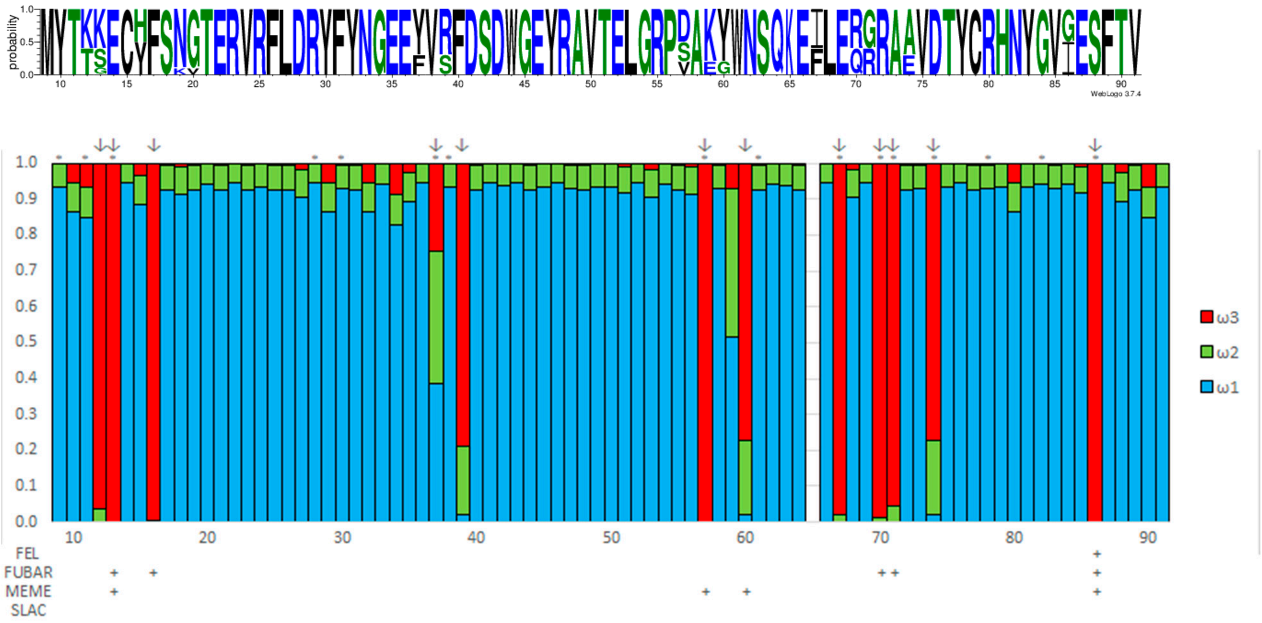

Figure S3. Distribution of positively selected sites in exon 2 DRB gene of European roe deer estimated by EasyCodeML (model 2). Red columns indicate the class of sites with a high probability of ω>1. In this model, ω1 = 0.06 and applies to ~60% of the codons (blue); ω2 = 1 at ~26% of the sites (green); and ω3 = 12.07 at ~14% of the sites (red); deletion in codon 65 (white). Diversity of the observed peptides with probabilities is indicated by a sequence conservation logo in upper part of the figure.
Antigen-binding codons (ABS) in the human ortholog [83] are indicated by asterisk, and positive selected sites in roe deer by arrows. Codons under selection are shown below the alignments and are indicated with + marks in respect to results from FEL, FUBAR, SLAC (for pervasive selection) and MEME (for episodic selection) methods performed in the Datamonkey 2.0 server.

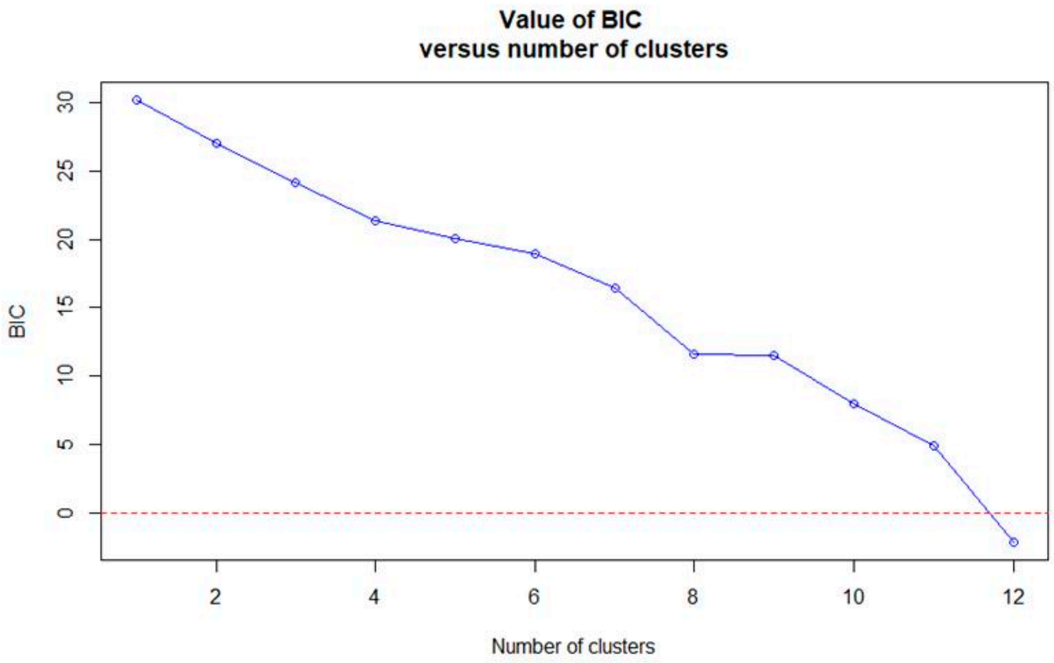

Figure S4. Choice of the number of clusters (K) for discriminant analysis of principle components (DAPC) for MHC supertypes in roe deer shows no point of stabilisation or 'elbow' at which the value begins to increase.

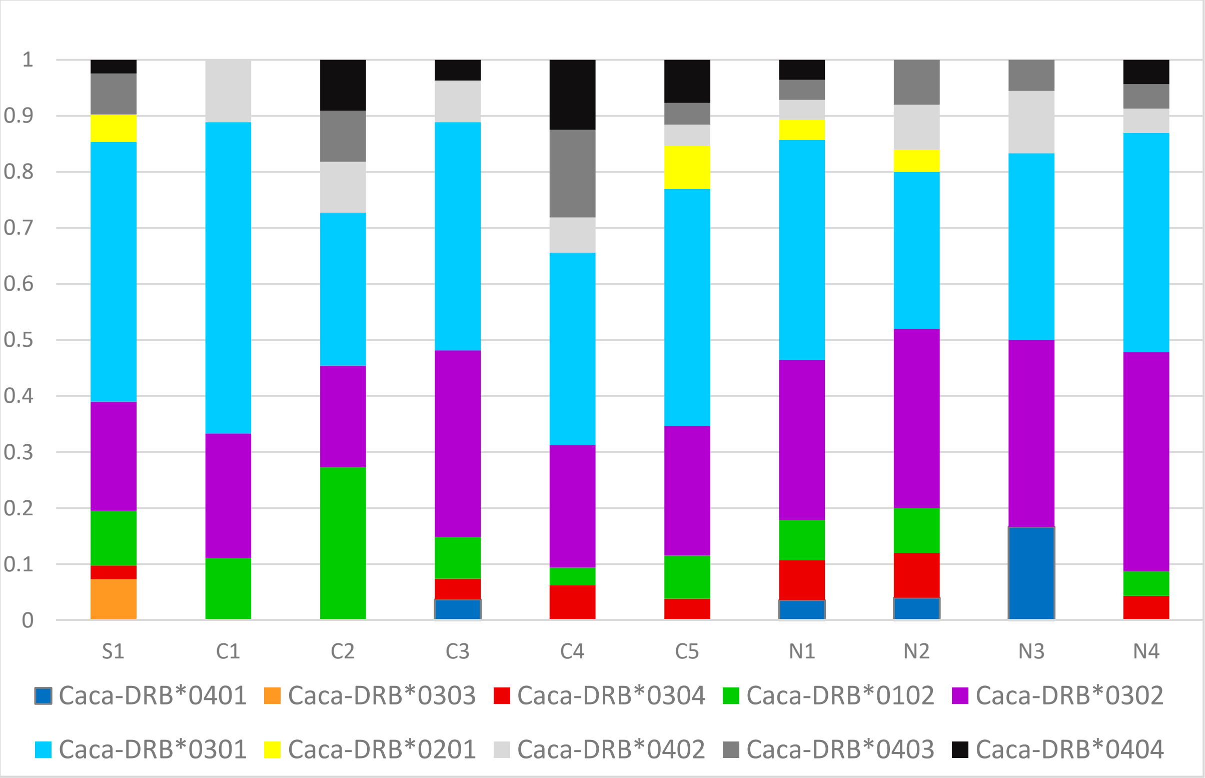

Figure S5. Plot of the frequency of MHC DRB exon 2 alleles in ten studied geographic groups (i.e., "populations") of European roe deer. The two most common alleles (Caca-DRB*301 and Caca-DRB*0302) were found in all groups/populations with different frequencies. Allele Caca-DRB*0301 (light blue) was present in 39% of individuals and Caca-DRB*0302 (violet) in 27% of individuals.
