# Supplementary material for: Neutral and Selective Processes Shape MHC Diversity in Roe Deer in Slovenia
Source: Animals (Basel). 2022 Mar 13;12(6):723. doi: 10.3390/ani12060723 (PMC8944837; doi:10.3390/ani12060723)
Supplement: Supplementary file 1 [file animals-12-00723-s001.zip › animals-1475938_Supplementary material_Tables.pdf]

Supplementary material. Tables

Table S1. Basic data on European roe deer females included in the study. All individuals were sampled in the period 2013–2015 in 58 hunting grounds continuously distributed throughout Slovenia (see Figure 1) and were previously included in determination of population genetic structure [44]. Membership of individuals in each of the geographical groups and the three genetic K-clusters, calculated by using neutral genetic markers (*ibid.*), is indicated.

Sample ID	Area	Group ("population")	Genetic cluster	Latitude	Longitude	MHC alleles	Structure Q-values
LME134	Coastal Slovenia (Kras and Istra)	S1	South-west	45.853	13.727	Caca-DRB*0102	Q1=0.796 Q2=0.121 Q3=0.082
LME139	Coastal Slovenia (Kras and Istra)	S1	South-west	45.746	13.896	Caca-DRB*0304	Q1=0.701 Q2=0.180 Q3=0.118
LME140	Coastal Slovenia (Kras and Istra)	S1	South-west	46.085	13.631	Caca-DRB*0302 Caca-DRB*0403	Q1=0.733 Q2=0.171 Q3=0.095
LME143	Coastal Slovenia (Kras and Istra)	S1	South-west	45.756	13.922	Caca-DRB*0301	Q1=0.730 Q2=0.140 Q3=0.130
LME252	Coastal Slovenia (Kras and Istra)	S1	South-west	45.738	13.948	Caca-DRB*0302 Caca-DRB*0301	Q1=0.758 Q2=0.134 Q3=0.108
LME253	Coastal Slovenia (Kras and Istra)	S1	South-west	45.738	13.948	Caca-DRB*0301	Q1=0.729 Q2=0.148 Q3=0.123
LME254	Coastal Slovenia (Kras and Istra)	S1	South-west	45.764	13.896	Caca-DRB*0302	Q1=0.793 Q2=0.120 Q3=0.087
LME1428	Coastal Slovenia (Kras and Istra)	S1	South-west	45.719	13.884	Caca-DRB*0301	Q1=0.700 Q2=0.161 Q3=0.139
LME1432	Coastal Slovenia (Kras and Istra)	S1	South-west	45.537	13.696	Caca-DRB*0301	Q1=0.731 Q2=0.159 Q3=0.110
LME1433	Coastal Slovenia (Kras and Istra)	S1	South-west	45.520	13.786	Caca-DRB*0302 Caca-DRB*0301	Q1=0.779 Q2=0.134 Q3=0.087
LME1438	Coastal Slovenia (Kras and Istra)	S1	South-west	45.835	13.740	Caca-DRB*0201	Q1=0.775 Q2=0.136 Q3=0.089
LME1440	Coastal Slovenia (Kras and Istra)	S1	South-west	45.806	13.612	Caca-DRB*0102 Caca-DRB*0403	Q1=0.73 Q2=0.137 Q3=0.133
LME1442	Coastal Slovenia (Kras and Istra)	S1	South-west	45.826	13.753	Caca-DRB*0201	Q1=0.799 Q2=0.124 Q3=0.077
LME1443	Coastal Slovenia (Kras and Istra)	S1	South-west	45.746	13.896	Caca-DRB*0301	Q1=0.709 Q2=0.159 Q3=0.131
LME1444	Coastal Slovenia (Kras and Istra)	S1	South-west	45.737	13.897	Caca-DRB*0301	Q1=0.630 Q2=0.198 Q3=0.172
LME1445	Coastal Slovenia (Kras and Istra)	S1	South-west	45.665	13.885	Caca-DRB*0303 Caca-DRB*0301	Q1=0.645 Q2=0.188 Q3=0.167
LME1449	Coastal Slovenia (Kras and Istra)	S1	South-west	45.592	13.758	Caca-DRB*0301	Q1=0.778 Q2=0.136 Q3=0.086
LME1450	Coastal Slovenia (Kras and Istra)	S1	South-west	45.537	13.696	Caca-DRB*0303 Caca-DRB*0301	Q1=0.755 Q2=0.133 Q3=0.110
LME1454	Coastal Slovenia (Kras and Istra)	S1	South-west	45.879	13.701	Caca-DRB*0302 Caca-DRB*0403	Q1=0.701 Q2=0.186 Q3=0.113

Sample ID	Area	Group ("population")	Genetic cluster	Latitude	Longitude	MHC alleles	Structure Q-values
LME1455	Coastal Slovenia (Kras and Istra)	S1	South-west	45.888	13.649	Caca-DRB*0302	Q1=0.701 Q2=0.163 Q3=0.136
LME1466	Coastal Slovenia (Kras and Istra)	S1	South-west	45.592	13.720	Caca-DRB*0302 Caca-DRB*0301	Q1=0.772 Q2=0.134 Q3=0.094
LME1467	Coastal Slovenia (Kras and Istra)	S1	South-west	45.547	13.785	Caca-DRB*0301	Q1=0.738 Q2=0.150 Q3=0.112
LME1468	Coastal Slovenia (Kras and Istra)	S1	South-west	45.592	13.720	Caca-DRB*0102 Caca-DRB*0301	Q1=0.769 Q2=0.145 Q3=0.086
LME1469	Coastal Slovenia (Kras and Istra)	S1	South-west	45.565	13.772	Caca-DRB*0303 Caca-DRB*0301	Q1=0.765 Q2=0.148 Q3=0.087
LME1470	Coastal Slovenia (Kras and Istra)	S1	South-west	45.547	13.785	Caca-DRB*0301	Q1=0.772 Q2=0.126 Q3=0.102
LME1471	Coastal Slovenia (Kras and Istra)	S1	South-west	45.729	13.935	Caca-DRB*0301	Q1=0.796 Q2=0.124 Q3=0.080
LME1472	Coastal Slovenia (Kras and Istra)	S1	South-west	45.765	13.935	Caca-DRB*0302 Caca-DRB*0301	Q1=0.788 Q2=0.131 Q3=0.081
LME1473	Coastal Slovenia (Kras and Istra)	S1	South-west	45.720	13.948	Caca-DRB*0404	Q1=0.736 Q2=0.165 Q3=0.098
LME1477	Coastal Slovenia (Kras and Istra)	S1	South-west	45.520	13.734	Caca-DRB*0102 Caca-DRB*0301	Q1=0.700 Q2=0.185 Q3=0.115
LME273	Julian Alps	C1	Central	46.396	14.104	Caca-DRB*0301 Caca-DRB*0402	Q1=0.516 Q2=0.267 Q3=0.217
LME274	Julian Alps	C1	Central	46.351	14.105	Caca-DRB*0302 Caca-DRB*0301	Q1=0.503 Q2=0.255 Q3=0.242
LME275	Julian Alps	C1	Central	46.369	14.079	Caca-DRB*0302 Caca-DRB*0301	Q1=0.550 Q2=0.250 Q3=0.199
LME276	Julian Alps	C1	Central	46.378	14.118	Caca-DRB*0102 Caca-DRB*0301	Q1=0.594 Q2=0.250 Q3=0.155
LME1476	Julian Alps	C1	Central	46.458	13.947	Caca-DRB*0301	Q1=0.511 Q2=0.261 Q3=0.228
LME122	Polhograjsko and Škofjeloško hills	C2	Central	46.137	14.458	Caca-DRB*0102 Caca-DRB*0302	Q1=0.363 Q2=0.365 Q3=0.272
LME123	Polhograjsko and Škofjeloško hills	C2	Central	46.182	14.445	Caca-DRB*0301	Q1=0.313 Q2=0.401 Q3=0.284
LME124	Polhograjsko and Škofjeloško hills	C2	Central	46.137	14.484	Caca-DRB*0302 Caca-DRB*0403	Q1=0.412 Q2=0.366 Q3=0.222
LME230	Polhograjsko and Škofjeloško hills	C2	Central	46.117	14.096	Caca-DRB*0102 Caca-DRB*0404	Q1=0.400 Q2=0.371 Q3=0.229
LME231	Polhograjsko and Škofjeloško hills	C2	Central	46.073	14.187	Caca-DRB*0301 Caca-DRB*0402	Q1=0.402 Q2=0.364 Q3=0.234
LME236	Polhograjsko and Škofjeloško hills	C2	Central	46.081	14.148	Caca-DRB*0102 Caca-DRB*0301	Q1=0.350 Q2=0.367 Q3=0.283

Sample ID	Area	Group (“population”)	Genetic cluster	Latitude	Longitude	MHC alleles	Structure Q-values
LME82	Dinaric Mountains	C3	Central	45.662	15.079	Caca-DRB*0301	Q1=0.309<br>Q2=0.323<br>Q3=0.368
LME93	Dinaric Mountains	C3	Central	45.779	14.809	Caca-DRB*0302	Q1=0.393<br>Q2=0.285<br>Q3=0.322
LME95	Dinaric Mountains	C3	Central	45.707	15.040	Caca-DRB*0301	Q1=0.320<br>Q2=0.314<br>Q3=0.364
LME125	Dinaric Mountains	C3	Central	45.939	14.344	Caca-DRB*0404	Q1=0.317<br>Q2=0.318<br>Q3=0.365
LME126	Dinaric Mountains	C3	Central	45.948	14.331	Caca-DRB*0304	Q1=0.315<br>Q2=0.388<br>Q3=0.297
LME136	Dinaric Mountains	C3	Central	45.509	15.053	Caca-DRB*0301	Q1=0.284<br>Q2=0.362<br>Q3=0.354
LME142	Dinaric Mountains	C3	Central	46.021	14.511	Caca-DRB*0301	Q1=0.383<br>Q2=0.306<br>Q3=0.309
LME164	Dinaric Mountains	C3	Central	45.644	15.002	Caca-DRB*0301	Q1=0.293<br>Q2=0.334<br>Q3=0.373
LME165	Dinaric Mountains	C3	Central	45.590	15.079	Caca-DRB*0302<br>Caca-DRB*0301	Q1=0.341<br>Q2=0.334<br>Q3=0.325
LME166	Dinaric Mountains	C3	Central	45.563	15.066	Caca-DRB*0301	Q1=0.343<br>Q2=0.357<br>Q3=0.299
LME168	Dinaric Mountains	C3	Central	45.716	15.066	Caca-DRB*0302	Q1=0.384<br>Q2=0.348<br>Q3=0.268
LME175	Dinaric Mountains	C3	Central	45.742	14.616	Caca-DRB*0302	Q1=0.345<br>Q2=0.332<br>Q3=0.323
LME176	Dinaric Mountains	C3	Central	45.760	14.667	Caca-DRB*0302	Q1=0.363<br>Q2=0.300<br>Q3=0.336
LME318	Dinaric Mountains	C3	Central	45.930	14.318	Caca-DRB*0302<br>Caca-DRB*0402	Q1=0.307<br>Q2=0.388<br>Q3=0.303
LME320	Dinaric Mountains	C3	Central	45.660	14.437	Caca-DRB*0301	Q1=0.359<br>Q2=0.309<br>Q3=0.331
LME321	Dinaric Mountains	C3	Central	45.779	14.834	Caca-DRB*0302<br>Caca-DRB*0402	Q1=0.407<br>Q2=0.321<br>Q3=0.270
LME322	Dinaric Mountains	C3	Central	45.644	14.771	Caca-DRB*0301	Q1=0.369<br>Q2=0.308<br>Q3=0.322
LME323	Dinaric Mountains	C3	Central	45.635	14.912	Caca-DRB*0302<br>Caca-DRB*0301	Q1=0.399<br>Q2=0.333<br>Q3=0.268
LME324	Dinaric Mountains	C3	Central	45.644	15.104	Caca-DRB*0302<br>Caca-DRB*0301	Q1=0.243<br>Q2=0.332<br>Q3=0.425
LME325	Dinaric Mountains	C3	Central	45.599	15.091	Caca-DRB*0401<br>Caca-DRB*0102	Q1=0.397<br>Q2=0.293<br>Q3=0.310
LME326	Dinaric Mountains	C3	Central	45.554	14.797	Caca-DRB*0102	Q1=0.511<br>Q2=0.264<br>Q3=0.225

Sample ID	Area	Group ("population")	Genetic cluster	Latitude	Longitude	MHC alleles	Structure Q-values
LME97	Kamniško-Savinjske Alps	C4	Central	46.273	14.820	Caca-DRB*0102<br>Caca-DRB*0301	Q1=0.219<br>Q2=0.473<br>Q3=0.308
LME104	Kamniško-Savinjske Alps	C4	Central	46.371	14.352	Caca-DRB*0304<br>Caca-DRB*0402	Q1=0.239<br>Q2=0.450<br>Q3=0.311
LME105	Kamniško-Savinjske Alps	C4	Central	46.398	14.378	Caca-DRB*0301	Q1=0.231<br>Q2=0.441<br>Q3=0.328
LME106	Kamniško-Savinjske Alps	C4	Central	46.390	14.651	Caca-DRB*0404	Q1=0.251<br>Q2=0.429<br>Q3=0.320
LME107	Kamniško-Savinjske Alps	C4	Central	46.399	14.677	Caca-DRB*0404	Q1=0.212<br>Q2=0.455<br>Q3=0.333
LME108	Kamniško-Savinjske Alps	C4	Central	46.273	14.612	Caca-DRB*0301	Q1=0.260<br>Q2=0.457<br>Q3=0.283
LME113	Kamniško-Savinjske Alps	C4	Central	46.229	14.963	Caca-DRB*0301	Q1=0.172<br>Q2=0.454<br>Q3=0.372
LME145	Kamniško-Savinjske Alps	C4	Central	46.408	14.651	Caca-DRB*0302<br>Caca-DRB*0404	Q1=0.201<br>Q2=0.481<br>Q3=0.318
LME147	Kamniško-Savinjske Alps	C4	Central	46.425	14.351	Caca-DRB*0301<br>Caca-DRB*0403	Q1=0.230<br>Q2=0.457<br>Q3=0.311
LME171	Kamniško-Savinjske Alps	C4	Central	46.281	14.444	Caca-DRB*0302	Q1=0.236<br>Q2=0.464<br>Q3=0.300
LME194	Kamniško-Savinjske Alps	C4	Central	46.264	14.729	Caca-DRB*0301<br>Caca-DRB*0402	Q1=0.202<br>Q2=0.475<br>Q3=0.323
LME196	Kamniško-Savinjske Alps	C4	Central	46.300	14.820	Caca-DRB*0302<br>Caca-DRB*0301	Q1=0.220<br>Q2=0.463<br>Q3=0.315
LME198	Kamniško-Savinjske Alps	C4	Central	46.174	14.678	Caca-DRB*0304<br>Caca-DRB*0301	Q1=0.249<br>Q2=0.452<br>Q3=0.298
LME200	Kamniško-Savinjske Alps	C4	Central	46.165	14.717	Caca-DRB*0302<br>Caca-DRB*0403	Q1=0.187<br>Q2=0.476<br>Q3=0.337
LME207	Kamniško-Savinjske Alps	C4	Central	46.363	14.742	Caca-DRB*0302<br>Caca-DRB*0301	Q1=0.229<br>Q2=0.453<br>Q3=0.318
LME242	Kamniško-Savinjske Alps	C4	Central	46.165	14.665	Caca-DRB*0302	Q1=0.269<br>Q2=0.430<br>Q3=0.301
LME305	Kamniško-Savinjske Alps	C4	Central	46.282	14.781	Caca-DRB*0301<br>Caca-DRB*0403	Q1=0.192<br>Q2=0.481<br>Q3=0.327
LME306	Kamniško-Savinjske Alps	C4	Central	46.301	14.859	Caca-DRB*0301<br>Caca-DRB*0403	Q1=0.260<br>Q2=0.435<br>Q3=0.305
LME309	Kamniško-Savinjske Alps	C4	Central	46.327	14.859	Caca-DRB*0302<br>Caca-DRB*0403	Q1=0.230<br>Q2=0.471<br>Q3=0.299
LME310	Kamniško-Savinjske Alps	C4	Central	46.309	14.742	Caca-DRB*0404	Q1=0.227<br>Q2=0.450<br>Q3=0.319
LME1	Posavsko hills	C5	Central	46.353	15.034	Caca-DRB*0301<br>Caca-DRB*0404	Q1=0.448<br>Q2=0.275<br>Q3=0.276

Sample ID	Area	Group ("population")	Genetic cluster	Latitude	Longitude	MHC alleles	Structure Q-values
LME3	Posavsko hills	C5	Central	46.319	15.051	Caca-DRB*0302<br>Caca-DRB*0201	Q1=0.416<br>Q2=0.312<br>Q3=0.272
LME5	Posavsko hills	C5	Central	46.340	15.009	Caca-DRB*0102<br>Caca-DRB*0301	Q1=0.327<br>Q2=0.317<br>Q3=0.355
LME6	Posavsko hills	C5	Central	46.381	15.001	Caca-DRB*0403<br>Caca-DRB*0404	Q1=0.343<br>Q2=0.303<br>Q3=0.354
LME8	Posavsko hills	C5	Central	46.311	15.049	Caca-DRB*0301	Q1=0.326<br>Q2=0.318<br>Q3=0.356
LME12	Posavsko hills	C5	Central	46.332	15.010	Caca-DRB*0301	Q1=0.330<br>Q2=0.313<br>Q3=0.357
LME14	Posavsko hills	C5	Central	46.349	15.049	Caca-DRB*0301	Q1=0.324<br>Q2=0.323<br>Q3=0.353
LME15	Posavsko hills	C5	Central	46.350	15.006	Caca-DRB*0302<br>Caca-DRB*0301	Q1=0.416<br>Q2=0.280<br>Q3=0.295
LME19	Posavsko hills	C5	Central	46.350	15.056	Caca-DRB*0301	Q1=0.386<br>Q2=0.296<br>Q3=0.318
LME23	Posavsko hills	C5	Central	46.346	15.055	Caca-DRB*0102<br>Caca-DRB*0302	Q1=0.336<br>Q2=0.308<br>Q3=0.354
LME33	Posavsko hills	C5	Central	46.346	15.004	Caca-DRB*0302<br>Caca-DRB*0301	Q1=0.409<br>Q2=0.294<br>Q3=0.297
LME40	Posavsko hills	C5	Central	46.327	15.011	Caca-DRB*0301	Q1=0.410<br>Q2=0.307<br>Q3=0.282
LME42	Posavsko hills	C5	Central	46.326	15.038	Caca-DRB*0302<br>Caca-DRB*0201	Q1=0.457<br>Q2=0.264<br>Q3=0.277
LME47	Posavsko hills	C5	Central	46.366	15.004	Caca-DRB*0304<br>Caca-DRB*0402	Q1=0.455<br>Q2=0.294<br>Q3=0.250
LME51	Posavsko hills	C5	Central	46.308	15.048	Caca-DRB*0301	Q1=0.315<br>Q2=0.330<br>Q3=0.355
LME61	Posavsko hills	C5	Central	46.352	15.001	Caca-DRB*0302<br>Caca-DRB*0301	Q1=0.305<br>Q2=0.330<br>Q3=0.365
LME77	Pohorje	N1	North-east	46.408	15.340	Caca-DRB*0302<br>Caca-DRB*0301	Q1=0.085<br>Q2=0.417<br>Q3=0.498
LME79	Pohorje	N1	North-east	46.398	15.522	Caca-DRB*0301	Q1=0.108<br>Q2=0.442<br>Q3=0.450
LME87	Pohorje	N1	North-east	46.398	15.587	Caca-DRB*0401<br>Caca-DRB*0302	Q1=0.081<br>Q2=0.486<br>Q3=0.433
LME96	Pohorje	N1	North-east	46.498	15.432	Caca-DRB*0302	Q1=0.099<br>Q2=0.445<br>Q3=0.456
LME111	Pohorje	N1	North-east	46.579	15.015	Caca-DRB*0102<br>Caca-DRB*0403	Q1=0.100<br>Q2=0.463<br>Q3=0.436
LME115	Pohorje	N1	North-east	46.417	15.340	Caca-DRB*0302<br>Caca-DRB*0301	Q1=0.093<br>Q2=0.505<br>Q3=0.402

Sample ID	Area	Group ("population")	Genetic cluster	Latitude	Longitude	MHC alleles	Structure Q-values
LME116	Pohorje	N1	North-east	46.408	15.340	Caca-DRB*0302<br>Caca-DRB*0301	Q1=0.103<br>Q2=0.494<br>Q3=0.403
LME117	Pohorje	N1	North-east	46.408	15.327	Caca-DRB*0301	Q1=0.112<br>Q2=0.470<br>Q3=0.418
LME181	Pohorje	N1	North-east	46.444	15.236	Caca-DRB*0302<br>Caca-DRB*0301	Q1=0.078<br>Q2=0.449<br>Q3=0.471
LME182	Pohorje	N1	North-east	46.480	15.262	Caca-DRB*0304	Q1=0.098<br>Q2=0.463<br>Q3=0.437
LME238	Pohorje	N1	North-east	46.381	15.418	Caca-DRB*0304<br>Caca-DRB*0404	Q1=0.094<br>Q2=0.442<br>Q3=0.464
LME239	Pohorje	N1	North-east	46.354	15.469	Caca-DRB*0301	Q1=0.103<br>Q2=0.466<br>Q3=0.431
LME255	Pohorje	N1	North-east	46.597	14.976	Caca-DRB*0301<br>Caca-DRB*0402	Q1=0.088<br>Q2=0.439<br>Q3=0.471
LME256	Pohorje	N1	North-east	46.597	14.963	Caca-DRB*0302<br>Caca-DRB*0301	Q1=0.094<br>Q2=0.478<br>Q3=0.426
LME257	Pohorje	N1	North-east	46.606	14.963	Caca-DRB*0102<br>Caca-DRB*0301	Q1=0.085<br>Q2=0.456<br>Q3=0.457
LME258	Pohorje	N1	North-east	46.588	15.002	Caca-DRB*0201	Q1=0.105<br>Q2=0.470<br>Q3=0.425
LME290	Pohorje	N1	North-east	46.453	15.444	Caca-DRB*0302<br>Caca-DRB*0301	Q1=0.089<br>Q2=0.472<br>Q3=0.439
LME120	Sub-Pannonian region	N2	North-east	45.984	15.660	Caca-DRB*0201	Q1=0.133<br>Q2=0.494<br>Q3=0.373
LME141	Sub-Pannonian region	N2	North-east	45.921	15.582	Caca-DRB*0301	Q1=0.106<br>Q2=0.479<br>Q3=0.415
LME183	Sub-Pannonian region	N2	North-east	46.335	15.508	Caca-DRB*0401<br>Caca-DRB*0402	Q1=0.132<br>Q2=0.490<br>Q3=0.378
LME184	Sub-Pannonian region	N2	North-east	46.299	15.534	Caca-DRB*0302<br>Caca-DRB*0301	Q1=0.110<br>Q2=0.495<br>Q3=0.395
LME203	Sub-Pannonian region	N2	North-east	46.237	15.391	Caca-DRB*0302<br>Caca-DRB*0301	Q1=0.105<br>Q2=0.499<br>Q3=0.394
LME205	Sub-Pannonian region	N2	North-east	46.255	15.352	Caca-DRB*0302	Q1=0.182<br>Q2=0.459<br>Q3=0.359
LME241	Sub-Pannonian region	N2	North-east	46.084	15.467	Caca-DRB*0301<br>Caca-DRB*0403	Q1=0.110<br>Q2=0.466<br>Q3=0.422
LME243	Sub-Pannonian region	N2	North-east	46.011	15.647	Caca-DRB*0301<br>Caca-DRB*0402	Q1=0.110<br>Q2=0.500<br>Q3=0.389
LME244	Sub-Pannonian region	N2	North-east	45.993	15.621	Caca-DRB*0304	Q1=0.168<br>Q2=0.462<br>Q3=0.370
LME245	Sub-Pannonian region	N2	North-east	45.984	15.634	Caca-DRB*0302	Q1=0.140<br>Q2=0.463<br>Q3=0.395

Sample ID	Area	Group ("population")	Genetic cluster	Latitude	Longitude	MHC alleles	Structure Q-values
LME246	Sub-Pannonian region	N2	North-east	46.002	15.621	Caca-DRB*0304	Q1=0.128 Q2=0.463 Q3=0.409
LME261	Sub-Pannonian region	N2	North-east	45.851	15.195	Caca-DRB*0102 Caca-DRB*0302	Q1=0.139 Q2=0.476 Q3=0.383
LME262	Sub-Pannonian region	N2	North-east	45.877	15.272	Caca-DRB*0301	Q1=0.125 Q2=0.484 Q3=0.391
LME263	Sub-Pannonian region	N2	North-east	45.878	15.195	Caca-DRB*0102 Caca-DRB*0301	Q1=0.137 Q2=0.491 Q3=0.372
LME264	Sub-Pannonian region	N2	North-east	45.895	15.259	Caca-DRB*0302 Caca-DRB*0403	Q1=0.126 Q2=0.453 Q3=0.419
LME265	Sub-Pannonian region	N2	North-east	45.877	15.285	Caca-DRB*0302	Q1=0.211 Q2=0.447 Q3=0.341
LME270	Sub-Pannonian region	N2	North-east	46.237	15.417	Caca-DRB*0302	Q1=0.086 Q2=0.462 Q3=0.452
LME84	Podravje and Slovenske gorice	N3	North-east	46.622	15.707	Caca-DRB*0401 Caca-DRB*0301	Q1=0.051 Q2=0.500 Q3=0.449
LME85	Podravje and Slovenske gorice	N3	North-east	46.658	15.681	Caca-DRB*0302 Caca-DRB*0402	Q1=0.055 Q2=0.557 Q3=0.388
LME118	Podravje and Slovenske gorice	N3	North-east	46.503	16.018	Caca-DRB*0302 Caca-DRB*0301	Q1=0.043 Q2=0.513 Q3=0.444
LME131	Podravje and Slovenske gorice	N3	North-east	46.614	15.615	Caca-DRB*0301	Q1=0.045 Q2=0.522 Q3=0.433
LME133	Podravje and Slovenske gorice	N3	North-east	46.650	15.668	Caca-DRB*0301 Caca-DRB*0403	Q1=0.060 Q2=0.549 Q3=0.391
LME135	Podravje and Slovenske gorice	N3	North-east	46.384	16.250	Caca-DRB*0401 Caca-DRB*0302	Q1=0.048 Q2=0.558 Q3=0.394
LME211	Podravje and Slovenske gorice	N3	North-east	46.469	15.744	Caca-DRB*0302 Caca-DRB*0301	Q1=0.072 Q2=0.540 Q3=0.388
LME298	Podravje and Slovenske gorice	N3	North-east	46.466	16.121	Caca-DRB*0302 Caca-DRB*0402	Q1=0.070 Q2=0.533 Q3=0.397
LME300	Podravje and Slovenske gorice	N3	North-east	46.466	16.095	Caca-DRB*0401 Caca-DRB*0302	Q1=0.058 Q2=0.554 Q3=0.388
LME317	Podravje and Slovenske gorice	N3	North-east	46.668	15.668	Caca-DRB*0301	Q1=0.062 Q2=0.550 Q3=0.387
LME121	Prekmurje	N4	North-east	46.764	16.023	Caca-DRB*0302 Caca-DRB*0301	Q1=0.116 Q2=0.551 Q3=0.331
LME138	Prekmurje	N4	North-east	46.637	16.073	Caca-DRB*0102	Q1=0.132 Q2=0.534 Q3=0.334
LME151	Prekmurje	N4	North-east	46.555	16.241	Caca-DRB*0302 Caca-DRB*0301	Q1=0.114 Q2=0.539 Q3=0.347
LME153	Prekmurje	N4	North-east	46.582	16.176	Caca-DRB*0301 Caca-DRB*0402	Q1=0.106 Q2=0.552 Q3=0.342

Sample ID	Area	Group ("population")	Genetic cluster	Latitude	Longitude	MHC alleles	Structure Q-values
LME154	Prekmurje	N4	North-east	46.546	16.240	Caca-DRB*0302 Caca-DRB*0301	Q1=0.123 Q2=0.538 Q3=0.339
LME155	Prekmurje	N4	North-east	46.563	16.280	Caca-DRB*0302 Caca-DRB*0301	Q1=0.106 Q2=0.551 Q3=0.343
LME156	Prekmurje	N4	North-east	46.546	16.240	Caca-DRB*0302	Q1=0.120 Q2=0.524 Q3=0.354
LME281	Prekmurje	N4	North-east	46.573	16.202	Caca-DRB*0302 Caca-DRB*0301	Q1=0.121 Q2=0.542 Q3=0.337
LME282	Prekmurje	N4	North-east	46.563	16.306	Caca-DRB*0302 Caca-DRB*0301	Q1=0.134 Q2=0.525 Q3=0.341
LME284	Prekmurje	N4	North-east	46.563	16.332	Caca-DRB*0403	Q1=0.111 Q2=0.539 Q3=0.350
LME285	Prekmurje	N4	North-east	46.635	16.321	Caca-DRB*0302 Caca-DRB*0404	Q1=0.114 Q2=0.549 Q3=0.337
LME286	Prekmurje	N4	North-east	46.564	16.241	Caca-DRB*0304	Q1=0.147 Q2=0.526 Q3=0.327
LME287	Prekmurje	N4	North-east	46.834	16.234	Caca-DRB*0301	Q1=0.116 Q2=0.533 Q3=0.351
LME288	Prekmurje	N4	North-east	46.852	16.247	Caca-DRB*0302	Q1=0.130 Q2=0.539 Q3=0.331
LME289	Prekmurje	N4	North-east	46.834	16.168	Caca-DRB*0301	Q1=0.103 Q2=0.548 Q3=0.349

Table S2. MHC DRB exon 2 alleles of European roe deer obtained from GenBank.

Allele name	Accession number	References
Caca-DRB*0202	KM488222.1	[25]
Caca-DRB*0204	KM488221.1	[25]
Caca-DRB*0203	KM488220.1	[25]
Caca-DRB*0201	KM488219.1	[25]
Caca-DRB*0401	KM488218.1	[25]
Caca-DRB*0303	KM488216.1	[25]
Caca-DRB*0301	KM488217.1	[25]
Caca-DRB*0304	KM488215.1	[25]
Caca-DRB*0102	KM488214.1	[25]
Caca-DRB*0302	KM488213.1	[25]
Caca-DRB*0301	U90925.1	[34]
Caca-DRB*0201	U90924.1	[34]
Caca-DRB*0101	U90923.1	[34]

Table S3. MHC DRB exon 2 genetic diversity in roe deer across its distribution range in Slovenia.

Geographical area	Abbr.	n	A	AR	Ho	He	HW (p value)	π (SD)	θ	Tajima's D
Overall		156	10	10.000	0.529	0.739	<0.001	0.042 (0.002)	0.015	**2.263**
Coastal Slovenia (Kras and Istra)	S1	29	8	4.560	0.414	0.713	<0.001	0.046 (0.006)	0.022	0.915
Julian Alps	C1	5	6	4.000	0.800	0.644	1.000	0.045 (0.018)	0.030	-1.205
Polhograjsko and Škofjeloško hills	C2	6	7	5.480	0.833	0.849	0.915	0.047 (0.010)	0.030	1.764
Dinaric Mountains	C3	21	7	4.060	0.286	0.704	<0.001	0.050 (0.007)	0.023	0.377
Kamniško-Savinjske Alps	C4	20	10	4.750	0.600	0.795	0.044	0.046 (0.007)	0.022	1.122
Posavsko hills	C5	16	8	4.380	0.531	0.672	<0.001	0.047 (0.005)	0.021	1.285
Pohorje	N1	17	8	4.830	0.647	0.765	0.008	0.047 (0.005)	0.023	0.921
Sub-Pannonian region	N2	17	5	5.070	0.471	0.800	0.004	0.048 (0.006)	0.023	1.042
Podravje and Slovenske gorice	N3	10	7	4.150	0.800	0.753	0.599	0.039 (0.011)	0.022	0.968
Prekmurje	N4	15	8	4.350	0.533	0.740	0.004	0.046 (0.007)	0.024	-0.399

Notes: n – number of individuals; A – number of alleles; AR – allelic richness; Ho – observed heterozygosity; He – expected heterozygosity; HW – Hardy–Weinberg equilibrium; π – nucleotide diversity; θ – 4Nμ for autosomal genes of diploid organisms; Tajima-s D (value in bold is significant; $p<0.05$)

Table S4. Pairwise F_{ST} (below) and p values (above; after Bonferroni corrections) between roe deer geographic groups/populations in Slovenia based on MHC DRB exon 2.

Population	S1	C1	C2	C3	C4	C5	N1	N2	N3	N4
S1	-	0.775	0.234	0.586	0.279	0.874	0.955	0.721	0.243	0.532
C1	-0.039	-	0.270	0.829	0.198	0.568	0.658	0.550	0.324	0.775
C2	0.023	0.053	-	0.171	0.360	0.252	0.378	0.505	0.189	**0.018**
C3	-0.014	-0.057	0.053	-	0.207	0.532	0.793	0.748	0.523	0.937
C4	0.008	0.031	0.001	0.014	-	0.486	0.496	0.793	0.396	0.234
C5	-0.017	-0.025	0.012	-0.010	-0.001	-	0.982	0.685	0.171	0.324
N1	-0.025	-0.036	0.005	-0.025	-0.011	-0.026	-	0.982	0.270	0.667
N2	-0.016	-0.016	-0.010	-0.020	-0.023	-0.017	-0.032	-	0.649	0.523
N3	0.019	0.001	0.027	-0.013	0.005	0.022	0.000	-0.024	-	0.414
N4	-0.005	-0.049	**0.089**	-0.036	0.016	0.000	-0.017	-0.013	-0.085	-

Value in bold is significant.

Table S5. Parameters of analysis of molecular variance of MHC DRB exon 2 data in ten roe deer groups/populations (overall and within three K-clusters) in Slovenia.

Source of variation	d.f.	SS	Variance components	Percentage of variation	Fixation index	p value
Within populations	334	122.684	0.367	98.90	0.011	0.520
Among populations	9	4.130	0.003	0.74	0.007	0.518
Among K-clusters	2	1.660	0.005	1.23	0.012	0.095

Table S6. Genetic diversity in Slovenian roe deer populations based on microsatellite data.

Population	He ± SD	Ho ± SD	HW (p value)	A ± SD	AR ± SD
S1	0.663 ± 0.134	0.670 ± 0.149	0.136	6.091 ± 2.427	3.364 ± 1.027
C1	0.544 ± 0.140	0.618 ± 0.289	0.704	3.364 ± 1.027	3.747 ± 1.259
C2	0.595 ± 0.181	0.576 ± 0.228	0.866	4.000 ± 1.414	3.584 ± 1.288
C3	0.604 ± 0.182	0.570 ± 0.190	0.802	5.364 ± 2.203	3.880 ± 1.355
C4	0.618 ± 0.173	0.623 ± 0.236	0.585	6.000 ± 2.366	3.710 ± 1.251
C5	0.653 ± 0.141	0.662 ± 0.141	0.611	5.818 ± 2.676	3.672 ± 1.329
N1	0.606 ± 0.175	0.647 ± 0.204	0.818	5.455 ± 2.339	3.707 ± 1.456
N2	0.601 ± 0.210	0.610 ± 0.250	0.891	5.273 ± 2.494	3.748 ± 1.789
N3	0.584 ± 0.277	0.582 ± 0.260	0.191	4.727 ± 2.453	3.722 ± 1.408
N4	0.596 ± 0.198	0.575 ± 0.252	0.379	5.182 ± 2.442	3.884 ± 1.248

Notes: Standard deviations (SD) are for average values per locus. He: expected heterozygosity; Ho: observed heterozygosity; HW: Hardy–Weinberg equilibrium; A: number of alleles; AR: allelic richness (calculated by the rarefaction method for the lowest sample size n = 5).

Table S7. Genetic diversity in three Slovenian roe deer genetic clusters revealed by STRUCTURE based on microsatellite data.

Cluster	He ± SD	Ho ± SD	A ± SD	AR ± SD
South-western	0.663 ± 0.134	0.670 ± 0.149	6.091 ± 2.427	6.020 ± 2.396
Central	0.648 ± 0.160	0.624 ± 0.151	7.091 ± 2.982	6.127 ± 2.559
North-eastern	0.618 ± 0.202	0.607 ± 0.218	6.818 ± 2.714	6.086 ± 2.643

Notes: Standard deviations (SD) are for average values per locus. He: expected heterozygosity; Ho: observed heterozygosity; A: number of alleles; AR: allelic richness (calculated by the rarefaction method for the lowest sample size n = 29).

Table S8. Pairwise F_{ST} (below) and p values (above; after Bonferroni corrections) between roe deer geographic groups/populations in Slovenia based on microsatellite data.

Population	S1	C1	C2	C3	C4	C5	N1	N2	N3	N4
S1	-	0.504	<0.001	0.017	0.002	<0.001	<0.001	0.005	<0.001	<0.001
C1	-0.001	-	0.087	0.294	0.668	0.114	0.149	0.253	0.097	0.174
C2	**0.065**	0.043	-	0.014	0.006	<0.001	0.005	<0.001	0.001	<0.001
C3	**0.014**	0.010	**0.046**	-	0.256	<0.001	0.703	0.725	0.210	0.026
C4	**0.021**	-0.008	**0.040**	0.005	-	0.006	0.424	0.655	0.078	0.003
C5	**0.033**	0.018	**0.059**	**0.025**	**0.019**	-	0.052	0.017	0.098	<0.001
N1	**0.029**	0.015	**0.041**	-0.004	0.001	0.011	-	0.963	0.313	0.004
N2	**0.019**	0.011	**0.059**	-0.004	-0.003	**0.016**	-0.012	-	0.438	0.056
N3	**0.035**	0.028	**0.064**	0.010	0.017	0.013	0.004	0.001	-	0.279
N4	**0.032**	0.020	**0.069**	**0.021**	**0.035**	**0.031**	**0.026**	0.017	0.007	-

Values in bold are significant, indicating that we did not find differences in F_{ST} between these pairs of comparisons.

Table S9. Pairwise F_{ST} values between roe deer clusters in Slovenia based on microsatellite data.

	**South-western**	**Central**
**Central**	0.018	
**North-eastern**	0.026	0.005

Table S10. Parameters of analysis of molecular variance of microsatellite data in ten roe deer groups/populations (overall and within three K-clusters) in Slovenia.

**Source of variation**	**d.f.**	**SS**	**Variance components**	**Percentage of variation**	**Fixation index**	**p value**
Within populations	334	1170.425	3.582	97.8	0.022	<0.001
Among populations	7	36.716	0.058	1.62	0.016	<0.001
Among K-clusters	2	16.504	0.019	0.55	0.006	0.148
